# Recent Advances in Developing Treatments of Kaposi’s Sarcoma Herpesvirus-Related Diseases

**DOI:** 10.3390/v13091797

**Published:** 2021-09-09

**Authors:** Eleonora Naimo, Jasmin Zischke, Thomas F. Schulz

**Affiliations:** 1 Institute of Virology, Hannover Medical School, 30625 Hannover, Germany; naimo.eleonora@mh-hannover.de (E.N.); zischke.jasmin@mh-hannover.de (J.Z.); 2 German Centre for Infection Research, Hannover-Braunschweig Site, 38023 Braunschweig, Germany; 3 Cluster of Excellence 2155 RESIST, Institute of Virology, Hannover Medical School, 30625 Hannover, Germany

**Keywords:** KSHV, HHV-8, Kaposi’s sarcoma, multicentric Castleman’s disease, primary effusion lymphoma, DNA polymerase, LANA, TK (ORF21), PK (ORF36), ORF59, vFLIP, RTA, LANA, CRISPR-Cas9

## Abstract

Kaposi-sarcoma-associated herpesvirus (KSHV) or human herpesvirus 8 (HHV-8) is the causative agent of several malignancies, including Kaposi’s sarcoma (KS), primary effusion lymphoma (PEL), and multicentric Castleman’s disease (MCD). Active KSHV replication has also been associated with a pathological condition called KSHV inflammatory cytokine syndrome (KICS), and KSHV may play a role in rare cases of post-transplant polyclonal lymphoproliferative disorders. Several commonly used herpesviral DNA polymerase inhibitors are active against KSHV in tissue culture. Unfortunately, they are not always efficacious against KSHV-induced diseases. To improve the outcome for the patients, new therapeutics need to be developed, including treatment strategies that target either viral proteins or cellular pathways involved in tumor growth and/or supporting the viral life cycle. In this review, we summarize the most commonly established treatments against KSHV-related diseases and review recent developments and promising new compounds that are currently under investigation or on the way to clinical use.

## 1. Introduction

Kaposi’s sarcoma-associated herpesvirus (KSHV) is a double-stranded DNA virus, discovered in 1994 by Patrick Moore and Yuan Chang and classified as a human gamma2-herpesvirus [1]. KSHV is associated with three neoplastic diseases: Kaposi’s sarcoma (KS) [1], multicentric Castleman’s disease (MCD) [2] and primary effusion lymphoma (PEL) [3]; As reviewed elsewhere, it meets the epidemiological and basic research requirements for recognition as an oncogenic agent and has thus been classified as a class I human carcinogen by the International Agency for Research against Cancer (IARC) [4,5]. Additionally, KSHV has also been associated with a pathological condition called KSHV inflammatory cytokine syndrome (KICS) [6] and rare cases of post-transplant polyclonal lymphoproliferative disorders [7,8] as well as with cases of plasmablastic lymphoma emerging from MCD [9].

The histopathological features of KS include atypical vascularization and neoangiogenesis with extensive infiltrates of inflammatory cells and the proliferation of atypical endothelial spindle cells [10]. Based on epidemiological criteria, KS has been classified into four different forms that are histologically similar [11]. Moritz Kaposi described KS for the first time in 1872 as a rare tumor endemic in the Mediterranean area that mostly affected middle-aged and older men. This “classic” KS is characterized by indolent skin lesions and viscera are only rarely involved [12]. 

In East and Central Africa, a more aggressive form of KS (the “endemic African” KS) in HIV-negative patients involves internal organs and lymph nodes in children and adults [13,14]. The “iatrogenic” KS affects up to 25% (in KSHV endemic regions) of transplant recipients under immunosuppressive therapy who were previously infected with KSHV [15,16]. This form of KS often recedes after the interruption of the immunosuppressive therapy [17,18].

In the 1980s, following the emergence of HIV/AIDS, the AIDS-associated or epidemic KS became a common manifestation among HIV-infected people [19]. AIDS-KS is the most aggressive form of KS. It can involve the lungs and the gastrointestinal tract [20]. After the introduction of the antiretroviral combination therapy (ART) against HIV, the incidence of this variant of KS was significantly reduced [19,21,22,23,24].

Primary effusion lymphoma (PEL) or body cavity-based lymphoma (BCBL) is a B cell non-Hodgkin lymphoma characterized by lymphomatous effusions in the pleural, pericardial and peritoneal body cavities [25]. PEL often arises in HIV-infected individuals and it is estimated to account for 2–5% of HIV-associated lymphomas [26]. PEL can also occur in transplant recipients [27]. KSHV DNA detection in the tumor cells is a diagnostic criterion for PEL [28,29].

Multicentric Castleman’s disease is characterized by systemic inflammation, increased levels of cytokines IL-6, IL-10 and vIL6 and by clinical symptoms like lymphadenopathy, fever, diarrhea and weight loss. The patients with MCD are more prone to non-Hodgkin lymphomas and organ failure [30,31,32,33].

KSHV replication in KSHV-HIV positive patients may induce a systemic inflammation characterized by high levels of IL-6 and IL-10 and high KSHV viral load in the blood. This non-malignant clinical manifestation is called KSHV associated inflammatory cytokine syndrome (KICS). As the clinical symptoms and laboratory abnormalities overlap with those seen in MCD, the diagnosis of KICS is predicated on the absence of the lymphadenopathy seen in MCD. KICS can also arise in KS or PEL patients and is often associated with a more severe disease course [6,34]. 

Current antiviral treatments against KSHV mainly rely on herpesviral DNA polymerase inhibitors. Although some of them efficiently inhibit KSHV replication in tissue culture, their efficacy against KSHV-associated disease is limited. Surgery, radiotherapy, and chemotherapy combined with antiviral agents and immunomodulatory molecules are used to obtain the best outcome for these patients. In trying to develop novel approaches to an effective pharmacological treatment of KSHV-associated diseases, either the combination of antiviral drugs directed against different viral targets, or the targeting of cellular proteins that are required for viral persistence, replication (‘dependency factors’) or the growth of tumor cells are being explored.

## 2. KSHV DNA Polymerase Inhibitors 

Since the discovery of KSHV, nucleoside inhibitors of the viral DNA polymerase such as ganciclovir, cidofovir, foscarnet, brivudine, and adefovir have provided the most potent inhibitors of KSHV replication in tissue culture [35,36,37,38,39,40]. In patients, some studies reported ganciclovir, valganciclovir, valacyclovir, famciclovir, cidofovir or foscarnet to reduce the shedding of KSHV in oral samples or KSHV viral load in peripheral blood, while others failed to notice pronounced effects of these drugs in treated patients [41,42,43]. With few exceptions [44], herpesviral DNA polymerase inhibitors (foscarnet, cidofovir, ganciclovir, valganciclovir) were found to be largely ineffective when used to treat established KS lesions [45,46,47,48]. In addition to these drugs, which are already approved for clinical use against other herpesviruses, several new promising nucleoside inhibitors have been identified in preclinical studies but are not yet approved or available for clinical treatments (for more details, see: [37,40,49,50,51].

The disappointing efficacy of herpesviral DNA polymerase inhibitors against KS is likely related to the fact that they are nucleoside analogs that, except for foscarnet, need to be phosphorylated to become active drugs. The first phosphorylation step is mediated by KSHV-encoded kinases, while generation of the nucleoside di- and triphosphates is due to the action of cellular kinases [40]. There are two KSHV kinases capable of activating nucleoside prodrugs by phosphorylation: a Ser/Thr kinase (vPK) encoded by open reading frame (ORF) 36 and a thymidine kinase (TK) encoded by ORF21. Both viral kinases are only expressed during the lytic phase of the viral cycle, and may thus only be able to exert their function in the relatively small number of infected cells in KS tumors that undergo lytic replication (see below).

Genes for herpesviral TKs occur in alpha- and gammaherpesviruses, but not in betaherpesviruses. In alpha- and gammaherpesviruses, the TK homologs are conserved: UL23 of herpes simplex virus 1 and 2 (HSV-1/2), ORF36 of varicella-zoster virus (VZV), BXLF1 of Epstein-Barr virus (EBV), and ORF21 of KSHV (52). Herpesviral TKs differ in their capacity to phosphorylate nucleoside analogs. Pyrimidine nucleosides such as brivudine and azidothymidine (zidovudine, the anti-HIV nucleoside reverse transcriptase inhibitor) are efficiently phosphorylated by KSHV TK [40,52]. Instead, PK/ORF36 efficiently phosphorylates purine analogs like valganciclovir [53].

As in the case of KS, the use of these viral DNA polymerase inhibitors against PEL and MCD has also in most cases only produced unsatisfactory clinical results [41,54,55,56,57,58]. The only notable exception is the combination of high dose ganciclovir and zidovudine in patients with MCD, which has shown promising clinical response rates [59]. This drug regimen is based on the activation of the prodrug zidovudine to a toxic moiety by KSHV TK, in combination with the antiviral effects of ganciclovir [59].

The limited efficacy of the DNA polymerase inhibitors pushed the scientific community to identify other suitable targets with different molecular mechanisms to treat KSHV-associated disease.

## 3. Antivirals Targeting Other Steps in the Viral Life Cycle

KSHV, like the other members of the Herpesviridae family, has a biphasic life cycle characterized by distinct patterns of viral gene expression [60]. KSHV establishes a permanent infection that lasts for the entire life of the infected host. During the KSHV latent phase, the viral DNA is maintained as a circular episome in the infected cells, replicated in dividing cells together with the cellular DNA [61,62], and a few latency-associated genes are transcribed: these encode the latency-associated nuclear antigen (LANA, encoded by ORF73), the viral homolog of cyclin D (vCyc-D/ORF72), the viral homolog of the fas-associated death domain-like interleukin-1-β-converting enzyme (FLICE-) inhibitory protein (vFLIP/ORF71), Kaposin (A, B and C encoded by K12) and 25 mature microRNAs [63,64,65,66]. In order to produce new viral progeny, KSHV has to periodically reactivate from latency and to switch into the productive (‘lytic’) phase of its life cycle. During the immediate-early and early stages of productive KSHV infection, only a subset of lytic viral genes is expressed. Following the replication of the viral DNA, viral genes encoding viral structural proteins are switched on (‘late’ phase of the productive replication cycle) in order to allow the production of new viral progeny [67].

In KS and PEL, the majority of KSHV infected cells adopt the latent program; therefore, a considerable effort has been made to target latent viral proteins or cellular pathways in which they interfere.

LANA (latency-associated nuclear antigen) is expressed in all the latently KSHV-infected cells [35,68]. In PEL cells, decreasing LANA expression with shRNA [69], by treatment with glycyrrhizic acid [70] or HSP90 inhibitors [71] induces cell death. The gene-editing technique, CRISPR-Cas9, has also been used successfully in two studies to target KSHV LANA and to target KSHV latency [72,73]. These findings suggest that LANA may be a promising viral target to disrupt KSHV latency. Its C-terminal DNA-binding domain (DBD) binds the latent KSHV replication origin in the terminal repeat (TRs) region of the viral genome; this interaction ensures the viral genome replication and segregation during cell mitosis [62,74,75,76,77]. The structure of the LANA DBD alone and in complex with the viral latent replication origin has been solved [76,78], allowing Kirsch and colleagues to discover and optimize new small compounds able to inhibit the binding of LANA to viral DNA in the low micromolar range [79,80,81]. In addition, Mubritinib (TAK165) was identified as a potent inhibitor of LANA-DNA binding and strongly reduced living KSHV PEL cells in vitro and in vivo [82]. 

Another potential target of the latent KSHV cycle is the viral FLICE-inhibitory protein (vFLIP). vFLIP is a potent activator of the NF-kB pathway and counteracts Fas-induced apoptosis [83,84]. Silencing vFLIP using siRNA [85] or using NF-kB inhibitors such as Bay 11-7082 [83,86,87,88] induces PEL cell apoptosis, suggesting that vFLIP may also represent an attractive therapeutic target. In order to activate the NF-kB pathway, vFLIP directly interacts with IKKγ/NEMO, a key player in the canonical NF-kB pathway [85,89]. The structure of a fragment of the coiled-coil domains of IKKγ/NEMO in complex with vFLIP has been solved [90], which provided the basis for a structure-guided development of vFLIP inhibitors. A conformationally constrained, stapled IKKγ peptide derived from the IKKγ–vFLIP interaction site interferes with the binding of IKKγ to vFLIP and enhances apoptosis in PEL cell lines [91]. Also, a tertiary protein structure mimic of the vFLIP-interaction site in the IKKγ/NEMO helix was able to induce cell death in PEL cell lines and to delay tumor growth in a PEL xenograft mouse model [88]. These findings indicate that it may be feasible to develop small molecule inhibitors targeting the vFLIP-IKKγ/NEMO interaction and showing a therapeutic effect against some KSHV-associated diseases.

New approaches to target the productive (‘lytic’) phase of the KSHV life cycle have also been developed but remain at a preclinical stage. Among the viral immediate early proteins to be expressed early after lytic reactivation are RTA, K-bZIP, and pORF45, crucial regulatory proteins and/or transcription factors [92,93]. RTA (encoded by ORF50) is necessary and sufficient to trigger the KSHV lytic phase, thus it is called “the master of KSHV lytic-switch” [94]. Long and colleagues recently described the efficiency of Gallic acid (GA) to inhibit RTA transcriptional activity by preventing its binding to target promoters. GA induces apoptosis in a PEL cell line in a dose-dependent manner [95].

Another novel viral target is the KSHV protein encoded by ORF59. pORF59 is a homologue of the DNA polymerase-associated processivity factor, which occurs in pro- as well as eukaryotes and in all herpesviruses. By acting in concert with the KSHV DNA polymerase encoded by ORF9, it facilitates the elongation of newly synthesized viral DNA. The compound NSC373989 was shown to target the pORF9/pORF59 complex and to inhibit viral DNA synthesis in vitro as well as KSHV lytic reactivation in PEL cells [96]. An inhibitor of pORF59 could provide an alternative approach to inhibiting viral DNA replication during the lytic phase of the replication and could potentially be used in combination with established competitive DNA polymerase inhibitors.

The structural similarity between the RNAse H-like nucleotidyltransferase domain contained in the HIV integrase and the two single-strand DNA (ssDNA) binding proteins essential for herpesviral DNA replication allowed the identification of XZ45, an HIV integrase inhibitor, as a compound that also inhibits the replication of KSHV and other herpesviruses [97]. Raltegravir and Dolutegravir, two HIV integrase inhibitors approved for clinical use against HIV, were successfully tested in vitro for their inhibition of the KSHV large terminase subunit encoded by pORF29. The C-terminal domain of KSHV pORF29 also shows a high degree of similarity with RNAse H-like nucleotidyltransferases and its inhibition impairs KSHV lytic reactivation in tissue culture [98].

In the case of herpes simplex virus (HSV), Varicella-Zoster virus (VZV), and human cytomegalovirus (HCMV), structure-based drug design has been employed to target capsid proteins, capsid assembly, and DNA encapsidation and this approach has already shown promising results [99,100,101,102]. One compound, letermovir, targets the HCMV terminase and the incorporation of viral DNA into newly formed viral capsids and has been approved for clinical use in stem cell transplant recipients [103]. However, it is not active against KSHV or other human herpesviruses. For KSHV, inhibitors of the pORF17 scaffold/protease polyprotein, which transiently fills the newly assembled capsid and is then released by autoproteolytic cleavage to allow packaging of capsids with viral DNA [104], have been developed and some have shown potency in tissue culture [105,106,107]. Nelfinavir, an HIV protease inhibitor, has been shown by Gantt and colleagues to inhibit KSHV, HSV and HCMV replication. Whether the KSHV pORF17 scaffold/protease is the key target of nelfinavir however still remains unclear [108,109].

Attempts were also made to target viral glycoproteins expressed on the surface of KSHV infected cells such as K8.1 and gH, by using immunotoxins that bind to these viral proteins. These immunotoxins could induce cell death in KSHV-infected cells in tissue culture, and a combination with ganciclovir increased their effect [110,111].

An opposite approach to inhibiting the viral lytic replication cycle involves its activation, with the aiming of taking advantage of the cell death occurring in lytically reactivated cells. Different treatments aimed to induce the viral lytic cycle in latently KSHV-infected cells have been attempted. Treatment with histone deacetylase (HADAC) inhibitors like sodium butyrate (NaB/SB), 12-*O*-tetradecanoylphorbol-13-acetate (TPA), and trichostatin (TSA), with the DNA methyltransferase inhibitor 5-Azacytidine (5-AZaC) or with some African autochthonous plant extracts can induce the lytic reactivation in vitro [112,113,114,115]. The proteasome inhibitor bortezomib induces KSHV and EBV lytic reactivation and it was successfully used in a clinical trial in combination with ganciclovir to treat MCD [57]. Liang and colleagues used the CRISPR-Cas9 system to inhibit the expression of KSHV miRNAs in latently KSHV positive PEL cells. This alters the expression of the mature miRNAs and induces upregulation of the viral lytic genes [116]. Recently, the suberoyl bis-hydroxamic acid (SAHA) was selected for its ability to induce KSHV lytic reactivation and apoptosis in a dose-dependent manner in PEL cells, indicating its possible therapeutic use [117].

Another possible strategy to counteract KSHV infection could be inhibition of virus entry into the target cell. This has so far proved difficult, because KSHV entry is mediated through diverse receptors, depending on the cell type that is to be infected. Binding of KSHV to the cell surface is achieved by heparan sulfate-proteoglycans (HSPGs) and DC-SIGN and entry is mediated through Ephrin receptors (EPHA2, 4, 5 and 7), integrins (α3β1, αVβ3, αVβ5, and α5β1) and xCT (reviewed in [118]).

## 4. Cellular Targets to Inhibit KSHV Replication

Instead of targeting the virus replication directly by inhibiting a viral protein, there are several ways of inhibiting the virus by targeting cellular processes that are essential for the virus to survive. As viruses hijack the host cells and exploit the cell machinery, there are several cellular proteins that play an important role in the viral life cycle and that can be targeted pharmacologically. A potential drawback of this approach is that these cellular processes are also important for cellular functions and that the inhibition of these targets may be accompanied by side effects.

### 4.1. Kinase Inhibitors

In KSHV infected cells, cellular tyrosine kinases play an essential role during the KSHV life cycle. Therefore, targeting cellular receptor tyrosine kinases such as c-kit, PDGFR, VEGFR, and Eph2A for antiviral treatment was investigated in several clinical studies. Treatment of KS patients with imatinib, a c-abl tyrosine kinase inhibitor in clinical use for the treatment of chronic myelogenous leukemia, resulted in clinical and histological regression of KS lesions in some patients [119,120]. Furthermore, sorafenib, which inhibits the VEGF receptor and is used to treat several malignancies like hepatocellular carcinoma or kidney carcinoma, was shown in a case report to achieve a complete remission of KS lesions in one patient [121]. Unfortunately, sorafenib showed only moderate effects when used to treat KS in a phase 1b clinical trial [122].

Of the more than 20 currently available inhibitors of cellular tyrosine kinases, five compounds (dasatinib, ponatinib, bosutinib, gefitinib, and nilotinib) were shown to also inhibit KSHV thymidine kinase (TK/pORF21), which, in contrast to its name, acts as an efficient protein tyrosine kinase [52,123]. Dasatinib and ponatinib also strongly inhibited KSHV early viral gene expression and the production of new viral progeny in B, endothelial and epithelial cells, most likely as a result of the inhibition of cellular tyrosine kinases, and dasatinib inhibited the growth of KSHV-driven endothelial tumors in a mouse xenograft model [52]. UNC3810A, a small molecule inhibitor of the receptor tyrosine kinase Tyro3, a member of the Tyro3/Axl/Mer (TAM) family of tyrosine kinases that promote the proliferation and survival of several cancers, was shown to be a potent inhibitor of PEL cell growth in a mouse xenograft model [124].

In KSHV infected cells, mTOR, a cellular kinase that belongs to the phosphatidylinositol 3-kinase related family of protein kinases, plays a key role in the life cycle of KSHV by promoting the activation of mTOR downstream of the viral G-protein coupled receptor (GPCR) homologue vGPCR. Blocking mTOR activity by rapamycin (sirolimus) inhibited cell growth in cell culture and tumor growth in a KSHV related mouse model, whereas overstimulation of the mTOR pathway resulted in the opposite, showing that this pathway is important for KSHV [125,126]. Several mTOR inhibitors reduce the growth of PEL cell lines in tissue culture and/or in mouse xenograft models, in particular when combined with an AKT inhibitor [127,128,129]. An ATP-competitive inhibitor of mTOR, MLN0128 (sapanisertib), induces apoptosis in PEL cells and reduces the growth of PEL in a xenograft model at nanomolar IC50 concentrations and is still effective against doxorubicin- or rapamycin-resistant PEL cell clones [129].

Similarly, the ERK/MAPK pathway was shown to be crucial in KSHV induced pathogenesis [130]. Several experimental compounds, e.g., sangivamycin and capsaicin, as well as trametinib, a MEK1/2 inhibitor approved for clinical use, can inhibit ERK activation and KSHV reactivation, and/or are able to induce apoptosis in PEL cell lines, suggesting that they could perhaps be used for the treatment of PEL [52,131,132]. BI-D1870, a RSK1/2 inhibitor, suppresses KSHV lytic gene expression and virus production. The RSK1/2 serine/threonine kinase is activated by its interaction with KSHV pORF45 and a small peptide blocking this interaction has been shown to inhibit viral lytic gene expression and viral progeny formation [133]. Furthermore, crizotinib, an inhibitor of ALK and c-Met, suppresses the growth of PEL cells in a mouse xenograft model [134].

### 4.2. HSP90 and HSP70 Inhibitors

HSP90 is a molecular chaperone required for the correct folding of cellular proteins. HSP90 inhibitors have found use as anti-cancer drugs to treat several malignancies, including lung or prostate cancer. Several groups have shown that HSP90 is also involved in essential steps in the KSHV life cycle: vFLIP, a viral latent protein, is found in a complex containing IKKγ/NEMO and HSP90 [135]. HSP90 serves as a co-factor for MAPK activation and latent viral gene expression of KSHV, and the KSHV K1 protein was shown to bind HSP90ᵦ [89,136,137].

Several HSP90 inhibitors have been used in cell culture studies and mouse models of KSHV malignancies. In particular treating KSHV positive cells with different HSP90 inhibitors (PU-H71, AUY922, BIIB021, NVP-BEP800, or 17-DMAG) leads to the proteasomal degradation of LANA and Eph2A and inhibited cell growth as well as induced apoptosis in PEL cells [71,138]. Furthermore, PU-H71, BIIB021, and AUY922 also repressed tumor progression in xenograft mouse models [138,139,140]. These are encouraging data, but HSP90 inhibitors have not yet been tested in clinical trials against KSHV malignancies.

Several HSP70 chaperone family members are involved in the formation of KSHV nuclear replication and transcription compartments (RTCs) during the early stages of the KSHV lytic cycle, and a small molecule HSP70 inhibitor, VER-155008, blocked KSHV RTC formation [141]. Formation of KSHV RTCs also involves neddylation, and the neddylation inhibitor MLN 4924 induces apoptosis in PEL cells [142].

### 4.3. Other Cellular Targets

Another promising drug candidate for treating patients with KSHV malignancies is bortezomib, a proteasome inhibitor, which has been shown to promote the KSHV and EBV lytic cycle. Bortezomib activates JNK and induces autophagy and apoptosis in PEL cell lines [143,144] and in a xenograft mouse model [145]. Further clinical studies confirmed this drug as a potential treatment against AIDS-associated KS in a pilot trial study, AMC-063 [146] and in combination with ganciclovir against MCD in a case report [55].

Recently, Chen et al. could show that pemetrexed, an anti-cancer drug already in clinical use, is able to inhibit the lytic replication of KSHV by blocking the dTMP synthesis in infected cells [147].

Other cellular factors that are important for the KSHV replication machinery and which could be targeted pharmacologically include topoisomerase II: (+)-Rutamarin, a topoisomerase II inhibitor that efficiently inhibits KSHV lytic DNA replication in BCBL-1 cells [148].

Hypoxia and hypoxia-mediated signaling are important factors that drive KSHV replication and may play a role in KSHV-associated malignancies. Thus, hypoxia-inducible factors (HIFs), the first mediators of the cellular response to hypoxia, play a crucial role in KSHV induced tumors and have been shown to activate KSHV lytic replication [149]. HIF1α can directly activate RTA, LANA, as well as the ORF34-37 cluster of lytic genes [150,151]. A small molecule inhibitor of HIF1α, PX-478, was shown to achieve a significant inhibition of PEL cell growth in tissue culture, suggesting that HIF1α could be a suitable target for treating this disease [152].

Other cellular metabolic targets include the metabolic sensor SIRT1 that is functionally required for sustaining the proliferation and survival of PEL cells. Inhibition of SIRT with the inhibitor tenovin-6 induced cell cycle arrest and apoptosis in PEL cell culture and significantly extended the survival of mice in a murine PEL model [153].

Heme oxygenase 1 (HO-1) is highly expressed in KSHV-infected HUVECs cells. Targeting HO-1 by siRNA or by the chemical inhibitor SnPP induces cell death in KSHV-infected endothelial cells and inhibits their growth as tumors in nude mice [154].

However, with the exceptions mentioned above, most of these inhibitors directed at cellular targets have so far not been studied in clinical trials. Nevertheless, these findings show the potential of targeting cellular mechanisms for the treatment of KSHV-associated diseases.

## 5. Monoclonal Antibodies and Immunomodulatory Therapies

Several monoclonal antibodies have been used to treat KSHV-related diseases. One of the targets to have been explored in this manner is the vascular endothelial growth factor (VEGF). Bevacizumab, a humanized monoclonal antibody against VEGF-A, showed an overall response rate of 31% in patients with HIV-associated KS [155,156]. Furthermore, rituximab, a monoclonal antibody against CD20 that is widely used to treat several types of B-cell lymphoma and autoimmune diseases, is effective in clinical trials against multicentric Castleman’s disease, either alone or in combination with liposomal doxorubicin [157]. However, treatment with rituximab can also cause the progression of KS in these patients [158,159].

Tocilizumab, a monoclonal antibody directed against the inteleukin-6 receptor that is currently used to treat rheumatoid arthritis and other autoimmune diseases, has also been shown to be beneficial in treating MCD [160,161,162].

The combination of lenalidomide, an immunomodulatory drug, with arsenic trioxide (ATO), which is normally used to treat acute promyelocytic leukemia, has been reported to produce encouraging results when treating PEL in a xenograft mouse model. In this study, lenalidomide/ATO treatment decreased the proliferation of PEL cells and downregulated the expression of KSHV latent viral proteins. This was associated with less NF-kB expression and downregulation of IL-6 and IL-10 as well as the inhibition of VEGF and the induction of apoptosis [163].

Pomalidomide, another immunomodulatory drug, has been shown to act against KS in HIV-negative and HIV-positive people in a clinical Phase I/II trial. Pomalidomide induced an increase in ICAM-1 and B7-2 expression in PEL cells, thereby leading to T cell activation and NK cell-mediated killing of PEL cells, which makes pomalidomide a promising candidate for the treatment of KSHV related malignancies [164,165].

## 6. KSHV Tropism and Models to Study the Virus

KSHV shows a relatively strict species tropism for humans. In vivo, viral DNA has been detected in human B cells [166], macrophages [167], keratinocytes, endothelial cells [168,169] and epithelial cells [170]. In vitro, KSHV can infect a broad spectrum of different cells including epithelial cells, endothelial cells, keratinocytes, fibroblasts, B- and T-lymphocytes, monocytes, macrophages and dendritic cells [167,168,171,172]. Besides, KSHV can infect non-human cell lines such as owl monkey kidney cells, baby hamster kidney fibroblasts cells, Chinese hamster ovary cells and mouse fibroblasts [173].

The B-cell lines derived from PEL patients are used to study KSHV pathogenesis in vitro as they are able to maintain the viral genome in a latent state. In contrast, the endothelial spindle cells lose the virus after a few passages in cell culture [174].

KSHV-infected lymphatic endothelial cells (LECs) exploit a unique transcription program with the expression of latent and lytic genes, which differs from the latency program described in stably infected blood endothelial cells (HUVECs) [175,176], both models are used to characterize KSHV molecular mechanisms of action.

Because of its restricted species tropism, studying KSHV infection in vivo is not straightforward. There are mainly three ways of how KSHV infection can be studied in vivo (nicely reviewed in [177]): the first is to infect non-human primates like common marmosets with KSHV [178]. The second approach involves the use of KSHV related viruses, like murine herpesvirus 68 (MHV-68), rhesus rhadinovirus (RRV) [179] or herpesvirus saimiri (HVS) [180].

MHV-68 infects mice and is used as a model to mimic KSHV infection, because it has been shown that 90% of the MHV-68 genes have homologs to KSHV [181]. MHV-68 behaves like a natural persisting pathogen in mice but without showing the disease. Therefore, MHV-68 is often used to study immunological topics like immunevasion and infection control by T cells. Nevertheless, this model is not suitable to study host colonialization and viral reactivation nor can it be used to study KSHV related malignancies [182].

The third approach, which is becoming increasingly more important, is to use humanized mice. In a xenograft mouse model, human PEL derived cell lines are implanted into immunodeficient mice to establish a PEL like phenotype [182,183]. McHugh et al. described an animal model, showing that coinfection with EBV establishes persistent KSHV infection in B cells, resulting in a PEL like phenotype in these mice [184].

## 7. Conclusions

Immunocompromised patients and people living in KSHV-endemic areas are most likely to be affected by KSHV-induced diseases. Despite more than 25 years of research on KSHV, we still lack effective therapies to counteract KSHV infection, reactivation and pathogenicity (Table S1). However, progress has been made over recent years and both new viral as well as cellular ‘druggable’ targets have emerged. Some of the insights into KSHV pathogenesis that have been made over the last two decades have also laid the ground for the development of active compounds that may find use in other malignancies. As KSHV-related malignancies are not among the most common cancers and often occur in economically disadvantaged countries, the development of effective drugs against KSHV and KSHV-associated diseases faces the obstacle of a lack of interest on the part of the pharmaceutical industry. Focusing on mechanisms of pathogenicity that are shared between KSHV-related and other, more common, malignancies may open up a way forward to overcome this obstacle.

## Data Availability

Not Applicable.

## Supplementary Materials

The following are available online at https://www.mdpi.com/article/10.3390/v13091797/s1, Table S1: Therapies in clinical trials.
