# Supplementary material for: Recent Advances in Developing Treatments of Kaposi’s Sarcoma Herpesvirus-Related Diseases
Source: Viruses. 2021 Sep 9;13(9):1797. doi: 10.3390/v13091797 (PMC8473310; doi:10.3390/v13091797)
Supplement: Supplementary file 1 [file viruses-13-01797-s001.zip › viruses-1341555-supplementary.pdf]

Therapies in clinical trials	
**Compound**	**References**
Cidofovir	(Kedes and Ganem 1997)(Medveczky et al. 1997)(Neyts and De Clercq 1997)(Sergerie and Boivin 2003)
Ganciclovir	(Kedes and Ganem 1997)(Medveczky et al. 1997)(Neyts and De Clercq 1997)(Sergerie and Boivin 2003)
Foscarnet	(Kedes and Ganem 1997)(Medveczky et al. 1997)(Neyts and De Clercq 1997)(Sergerie and Boivin 2003)
Brivudine	(Beauclair et al. 2020)(Coen et al. 2014)
Adefovir	(Coen et al. 2014)
Imatinib	(Koon et al. 2005)(Koon et al. 2014)
Sorafenib	(Ardavanis et al. 2008) (Uldrick et al. 2017)
Rapamycin/Sirolimus	(Krown et al. 2012)(Sodhi et al. 2006) (Roy et al. 2013)(Sin et al. 2007)(Bhatt et al. 2010)
Bortezomib	(Granato et al. 2017)(Matta and Chaudhary 2005)(Sarosiek et al. 2010) (Reid et al. 2020)
Rituximab	(Uldrick et al. 2014)(Lurain et al. 2019)
Bevacizumab	(Uldrick et al. 2012)(Ablanedo-Terrazas et al. 2015)
Siltuximab	(van Rhee, Greenway, and Stone 2018) (Ramaswami et al. 2019)
Tolicizumab	(Ramaswami et al. 2020) (Nagao, Nakazawa, and Hanabusa 2014)(Aita et al. 2020)
Pomalidomide	(Polizzotto et al. 2016)
